# Conserved and Opposite Transcriptome Patterns during Germination in *Hordeum vulgare* and *Arabidopsis thaliana*

**DOI:** 10.3390/ijms21197404

**Published:** 2020-10-07

**Authors:** Yanqiao Zhu, Oliver Berkowitz, Jennifer Selinski, Andreas Hartmann, Reena Narsai, Yan Wang, Peisheng Mao, James Whelan

**Affiliations:** 1 Forage Seed Lab, Key Laboratory of Pratacultural Science, China Agricultural University, Beijing 100193, China; zhuyq@qibebt.ac.cn (Y.Z.); maopeisheng@hotmail.com (P.M.); 2 Department of Animal, Plant and Soil Science, School of Life Science, La Trobe University, Bundoora, Victoria 3086, Australia; O.Berkowitz@latrobe.edu.au (O.B.); jennifer.selinski@uni-bielefeld.de (J.S.); 18756804@students.latrobe.edu.au (A.H.); R.Narsai@latrobe.edu.au (R.N.); Yan.Wang@latrobe.edu.au (Y.W.); 3 Australian Research Council Centre of Excellence in Plant Energy Biology, La Trobe University, Bundoora, Victoria 3086, Australia

**Keywords:** *Arabidopsis thaliana*, *Hordeum vulgare*, mitochondria, energy metabolism photosynthesis, seed germination, transcriptome

## Abstract

Seed germination is a critical process for completion of the plant life cycle and for global food production. Comparing the germination transcriptomes of barley (*Hordeum vulgare*) to *Arabidopsis thaliana* revealed the overall pattern was conserved in terms of functional gene ontology; however, many oppositely responsive orthologous genes were identified. Conserved processes included a set of approximately 6000 genes that peaked early in germination and were enriched in processes associated with RNA metabolism, e.g., pentatricopeptide repeat (PPR)-containing proteins. Comparison of orthologous genes revealed more than 3000 orthogroups containing almost 4000 genes that displayed similar expression patterns including functions associated with mitochondrial tricarboxylic acid (TCA) cycle, carbohydrate and RNA/DNA metabolism, autophagy, protein modifications, and organellar function. Biochemical and proteomic analyses indicated mitochondrial biogenesis occurred early in germination, but detailed analyses revealed the timing involved in mitochondrial biogenesis may vary between species. More than 1800 orthogroups representing 2000 genes displayed opposite patterns in transcript abundance, representing functions of energy (carbohydrate) metabolism, photosynthesis, protein synthesis and degradation, and gene regulation. Differences in expression of basic-leucine zippers (bZIPs) and Apetala 2 (AP2)/ethylene-responsive element binding proteins (EREBPs) point to differences in regulatory processes at a high level, which provide opportunities to modify processes in order to enhance grain quality, germination, and storage as needed for different uses.

## 1. Introduction

Seed germination is the earliest process in the life cycle of flowering plants. The quiescent seed imparts flowering plants with the advantage of being able to withstand periods where growth and survival may not be possible. This may last from weeks to decades and in favorable conditions vegetative growth can recommence rapidly. The large reserves of proteins, lipids, and/or carbohydrates present in seeds not only provide the essential energy source to support germination until plants become autotrophic, but also deliver an estimated 70% of the calories consumed by humans [1]. Seeds change from being metabolically inert to displaying high rates of metabolic activity within hours, and for many species, seed germination can be completed in as little as 24 h, progressing from a dry seed to the radicle rupturing the seed coat [2]. 

The roles of gibberellic acid (GA) in promoting germination and abscisic acid (ABA) in maintaining dormancy have been extensively studied in the past [3,4]. The levels of ABA increase during seed maturation, inhibiting germination by preventing water uptake. GA promotes germination by binding to the GA receptor, which results in the degradation of DELLA proteins (aspartic acid–glutamic acid–leucine–leucine–alanine), strong repressors of germination [5]. Ultimately, this results in the activation of a series of transcription factors (TFs) that increase the levels of various hydrolytic enzymes necessary for germination to proceed. Although the overall roles of ABA and GA have been well studied, how germination is initiated is still actively investigated. Studies in *Arabidopsis thaliana* have shown that a distinct small number of cells in the dormant embryotic radicle are decision making. The cells initiating the ABA- and GA-dependent signaling are spatially separated, with transport of hormones from these separate locations ultimately leading to the break of dormancy [6]. In addition to ABA and GA, a variety of other hormones, including auxin and ethylene, likely play a role in germination via interacting signaling pathways [7].

Comparative analyses carried out for seed development in Arabidopsis, *Medicago trunculata*, soybean (*Glycine max*), maize (*Zea mays*), rice (*Oryza sativa*), wheat (*Triticum aestivum*), and barley have revealed conserved networks of genes regulated by the TFs FUSCA3 (*FUS3)*/ abscisic acid-insensitive protein 3 (*ABI3)*/LEAFY COTYLEDON1 (LEC1) [1]. In contrast, other networks are not conserved across monocots and dicots. LEC2 has only been found in dicot species while sucrose non-fermenting 1 (SNF1) is specific to monocots and has a role in the storage phase of endosperm development [1,8]. Several transcriptome studies are available for seed germination in Arabidopsis [9,10], rice [11], and barley [12]. The methodological diversity of these studies, using different microarray platforms and RNASeq, prompts caution when making comparisons. In Arabidopsis, one group of transcripts represents the breakdown of mRNAs from seed maturation, another group of genes displays a transient increase in transcript abundance early in germination between 1 and 6 h after imbibition, and other transcripts increase in abundance 6 h after imbibition [13,14]. It has been shown that the transcripts that display a transient increase are enriched in functions for organelle biogenesis in the earliest stages of germination. This drives the large increase in metabolic functions that are encoded by transcripts increasing 6 h after imbibition to establish autotrophic growth [13,14]. 

Examination of organelle morphology and activity of mitochondria in dry seeds of pea (*Pisum sativum* L.), sunflower (*Helianthus annuus*), maize, and peanut (*Arachis hypogaea*) revealed that they are active immediately on hydration and contain simple structures [15,16,17,18,19,20]. Upon hydration (phase I) respiration increases to an initial plateau (phase II) and a subsequent increase in phase III occurred after initiation of mitochondrial biogenesis. This sequence of events has been confirmed at a molecular level with examination of various proteins involved in mitochondrial respiratory activity [19,21,22,23]. A model of simple-structured mitochondria, termed pro-mitochondria, that become electron dense, develop cristae and mature into organelles displaying high rates of metabolic activity through the tricarboxylic acid (TCA) cycle, with the respiratory chain to provide ATP and drive growth being based on the early studies of light and heavy fractions of mitochondria being isolated [13,21,23,24]. This was supported by the observation that mitochondria isolated from seed just after imbibition are competent to import proteins, which requires a membrane potential generated by electron transport [21,25]. Further studies examining the mitochondrial genome after imbibition revealed that mitochondrial fusion followed by fission is important in generating the new generation of mitochondria [26]. The importance of mitochondria in germination is underscored by a study in *Brassica napus* that revealed changes in the abundance of metabolites associated with the mitochondrial TCA cycle, malate and aspartate, that are associated with the ability to mobilize reserves faster through mitochondrial metabolism [27]. A redox-mediated switch occurs upon imbibition to activate a latent basal mitochondrial activity as mitochondrial biogenesis cannot occur de novo [28].

Much progress has been made on understanding various aspects of seed biology, especially in Arabidopsis, with a greater understanding of the epigenetic, genetic, hormonal, and post-translational regulation of seed dormancy and germination, and importantly also on the interaction of the molecular components linking these processes and the environmental cues that trigger germination [29,30]. While transcriptome datasets covering seed germination are available for several species, no comprehensive comparison has been carried out on the changes in transcriptomes over the time span from imbibition to the completion of germination to determine the conserved and specific features of germination between Arabidopsis (oil storage, small seed) and barley grain (starch storage), which are evolutionary separated by over 140 million years [31]. To identify conserved and opposite features of barley seed germination, we carried out an in-depth transcriptome analysis of barley seed germination over a 48 h time course to compare with data from Arabidopsis [10].

## 2. Results

### 2.1. Physiological Changes during Barley Germination

**Figure 1:**
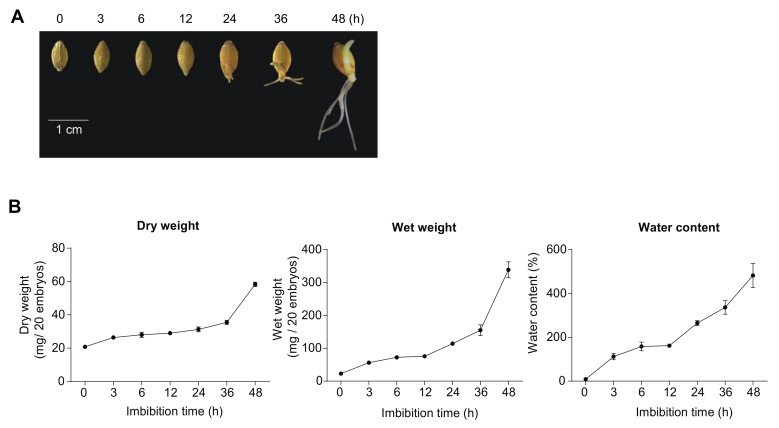
Barley grain germination. (**A**) Developmental stage of barley embryos that were analyzed. Barley embryos were manually dissected from dry (0 h) or imbibed (3 to 48 h) seeds to determine dry and wet weight, to isolate mitochondria and RNA, and for use in oxygen consumption measurements. (**B**) To determine the water content, we dissected embryos from barley grain 0 to 48 h after imbibition. Wet and dry weights were measured for barley embryos and the water content was calculated from these data. For each time point, the mean standard error is shown (*n* = 50). Both wet and dry weight increased steadily over time up until 36 h, when the radicle had emerged and thus germination had occurred. A larger increase in both wet and dry weight was observed after 36 h. Water content initially increased up until 6 h, plateaued, and continued steadily after 12 h.

Germination in barley was analyzed from dry grain (0 h) to 3, 6, 12, 24, 36, and 48 h after imbibition. The emergence of the radicle and coleoptile was observed after 24 h of imbibition (Figure 1A). The water content of barley embryos steadily increased over 48 h with an increase in wet weight observed until 36 h (from ≈20 mg to ≈180 mg). Thereafter, a large increase in wet weight was observed (from ≈180 mg to ≈370 mg). In contrast, the dry weight only increased less than twofold up to 36 h (from ≈20 mg to ≈35 mg), but thereafter showed a large increase from ≈35 mg to ≈60 mg (Figure 1B). For both the barley grain used in this study and the Arabidopsis seed from previous studies [10], the germination rate was greater than 99% and germination was complete after 24 h on the basis of the observation that the radicle had emerged (Figure 1A). 

### 2.2. Conserved Transcriptome Patterns during Germination

RNAseq analyses at seven time points for barley, i.e., dry grain (0 h) and 3, 6, 12, 24, 36, and 48 h after imbibition, revealed that out of a total of 81,279 protein-coding genes annotated in the barley genome release (IBSC v2) (Table S1A), a total of 21,506 differentially expressed genes (DEGs) were detected across all time points when compared to the dry seed (0 h) (Table S2A). A total of 2026 DEGs were differentially expressed at all time points post-imbibition compared to 0 h (Figure S1). A rapid change in the expression was also observed with 3535 DEGs at 6 to 48 h and 2360 DEGs at 12 to 48 h, while 3111 DEGs were specifically responsive in the three latest time points (24, 36, and 48 h; Figure S1), coinciding with radicle emergence at 24 h. This time point defined the completion of germination as the radicle had emerged and early seedling growth commenced (Figure 1). 

**Figure 2:**
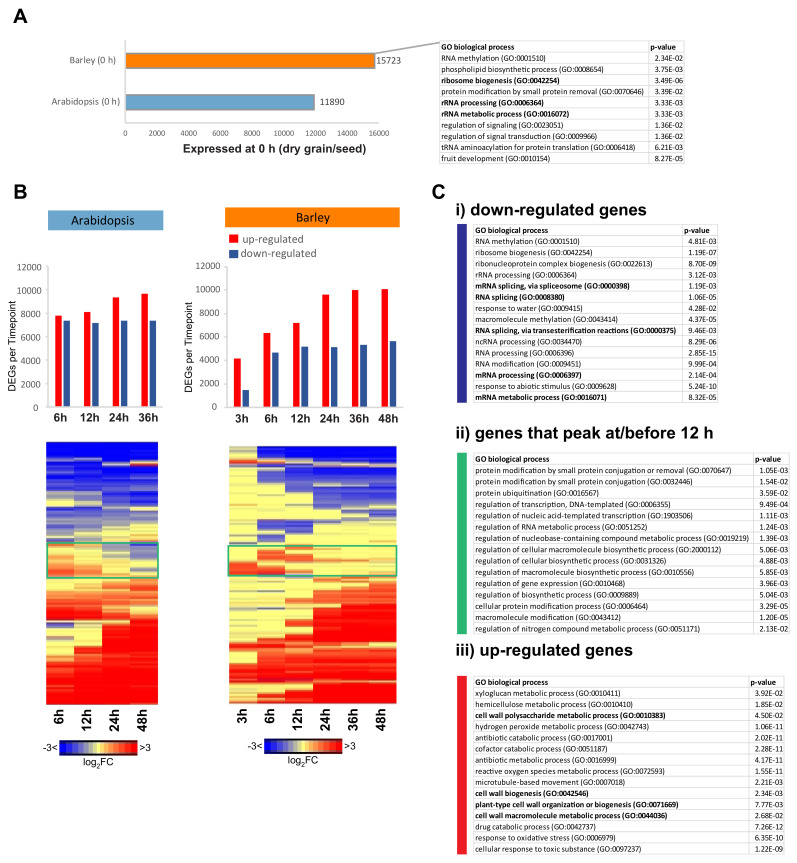
Transcriptome of barley grain germination. Differentially expressed genes (DEGs; |log_2_(fold-change)|>1, false discovery rate (FDR) < 0.05) during the course of germination were identified by comparing to the imbibed seed (0 h) using RNAseq. (**A**) The number of expressed genes at 0 h is shown, and those that overlapped with the Arabidopsis dry seed are indicated in bold. (**B**) The number of DEGs are shown for barely and Arabidopsis as well as the heatmaps following average linkage hierarchical clustering of the fold changes. Genes that peaked at 12 h or earlier during germination are indicated in the green boxes. (**C**) The top 15 Gene Ontology (GO) biological processes that were enriched in the (i) downregulated genes, (ii) genes that peaked at 12 h or earlier, and (iii) upregulated genes are shown. Categories are in order of fold enrichment. The functional categories that overlapped with the outputs following the same analysis in Arabidopsis are indicated in bold. All GO enrichment was carried out using Fisher’s test and Bonferroni FDR correction.

**Figure 3:**
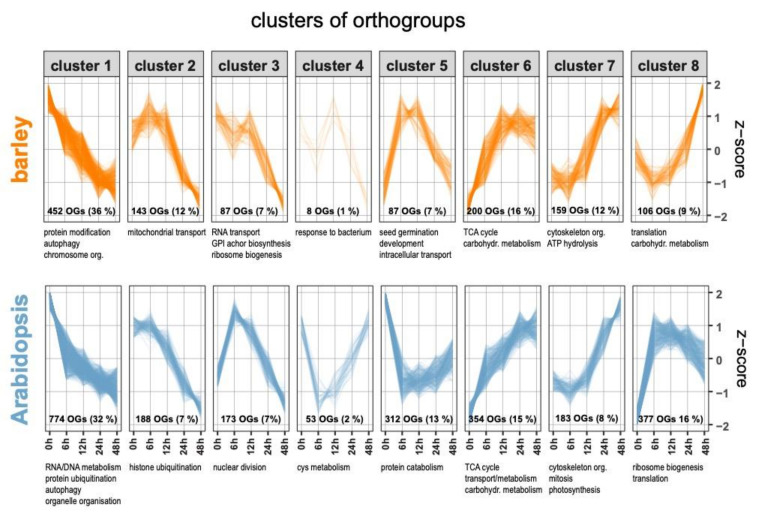
Comparison of transcript abundance profiles during germination in barley and Arabidopsis. For cross-species comparison of transcript abundance changes during germination, we compared changes in transcript abundance in barley to published data in Arabidopsis [10]. Genes in both species were classified into orthologous groups of genes (“orthogroups”) using OrthoFinder 2 [35] and clustered using the Clust algorithm [36]. A summary of the enriched GO terms for genes in each cluster is given below each panel, and the full list available in Table S6. The number in each panel gives the number of orthogroups in each cluster and the percentage of all orthogroups per species.

To compare changes in transcriptome patterns between barley and Arabidopsis during germination, we took three approaches. Firstly, given that a significant number of transcripts are present in dry grain/seed (0 h), the dry grain/seed transcriptomes were compared between barley and Arabidopsis (Figure 2A). Secondly, hierarchical clustering of the transcriptomes was carried out to determine if similar responses were occurring over time (Figure 2B,C), and thirdly, orthologous genes were defined to represent orthogroups that contain orthologues and paralogues, and their patterns of transcript abundance were compared (Figure 3). 

For the barley and Arabidopsis, 15,723 and 11,890 transcripts could be detected, respectively (Figure 2A, Table S1C). The enriched Gene Ontology (GO) terms of ribosomal biogenesis, rRNA processing, and rRNA metabolic process were in common between the two species (Figure 2C, Table S3A), which is consistent with findings that showed that these processes play an important role in germination [32,33,34]. Hierarchical clustering of the changes in gene expression defined three major groups of DEGs: (1) those that decreased in abundance; (2) a group of genes that initially increased in abundance up to 12 or 24 h post-imbibition and then decreased again, termed the transient group; and (3) those that increased in abundance (Figure 2B,C). The latter group represented genes encoding proteins that are required for germination and early seedling development and were enriched in the GO terms relating to cell wall biogenesis, organization, and metabolism, which are associated with growth (Figure 2Ciii, Table S3B). Transcripts that were downregulated in abundance included processes of RNA modification, possibly representing the degradation of RNA that was present from seed development and maturation (Figure 2Ci). For the transient group of genes, protein modification, mainly related to ubiquitination, and regulation of various processes associated with transcription and RNA synthesis were enriched (Figure 2Cii, Table S3B). An alternative approach using a self-organizing maps algorithm confirmed these results (Figure S2).

To carry out an unbiased comparison of germination at a transcriptome level between barley and Arabidopsis, we used OrthoFinder [35] to define sets of orthologous genes in the two species. These gene sets are termed orthogroups and contain both orthologues and paralogues. OrthoFinder takes into account protein length, as smaller proteins can be lost in BLAST searches due to the scoring system used in the algorithm. A total of 10,541 orthogroups were defined between Arabidopsis and barley, representing 25,714 genes in Arabidopsis and 26,983 genes in barley (Table S4). The orthologous genes between barley and Arabidopsis were subsequently analyzed with Clust [36] to define sets of orthologues genes that are tightly co-expressed within and across species. Compared to other approaches that assign all genes to clusters, Clust only allocates co-expressed genes/orthogroups to clusters and with low levels of dispersion compared with other partitioning methods while discarding the other genes/orthogroups. For barley and Arabidopsis, a total of 1242 and 2414 orthogroups, respectively, out of the total of 10,541 orthogroups were assigned to one of eight clusters. For each species, this analysis classified their orthogroups into six clusters that displayed a similar pattern in the two species (Figure 3, Table S5A,B). 

For clusters 1, 2, 6, and 7, co-expression of orthogroups was highly conserved between barley and Arabidopsis, while clusters 3 and 8 showed similar trends with somewhat different kinetics, and clusters 4 and 5 were contrasting in the two species. Subsequently, a GO term enrichment analysis was performed for the genes in the orthogroups included in each of the clusters (Table S6A,B). Overall, this showed similarity in significant GO terms for many of these clusters (clusters 1, 6, 7, 8), while some were divergent. This may have resulted from differences in the physiology and molecular processes of germination in barley (starch storage) and Arabidopsis (oil seed), leading to functional diversification of orthologous genes and corresponding differences in expression patterns, but also from availability and accuracy of GO term annotations for Arabidopsis (annotations for 22,365 of 27,655 coding genes, i.e., 80%) compared to barley (annotation for 16,542 of 39,841 genes annotated, i.e., 41%). Cluster 1 showed a decline in expression of genes in the orthogroups from the 0 h time point and clusters 2 and 3 between 6 h and 12 h (Figure 3). Genes in these three clusters were associated with enriched GO terms related to RNA/DNA metabolism, autophagy, protein modifications, and organellar function. These likely represent the breakdown of storage reserves and mRNA that remained after seed maturation. By contrast, orthogroups in clusters 6, 7, and 8 contain genes for which transcripts increase in abundance during germination. Orthogroups in clusters 6 and 7 showed very similar expression kinetics in both species, with genes in cluster 6 increasing from 0 h and peaking between 12 h and 24 h, while expression of genes in cluster 7 increased only after 6 h to 12 h after imbibition (Figure 3). GO terms enriched for genes in cluster 6 were largely representative for an increased metabolic activity (mitochondrial TCA cycle, carbohydrate metabolism) and genes in cluster 7 were related to GO terms representing increased growth (cytoskeleton organization, mitosis). Genes of orthogroups in cluster 8 increased their expression very quickly to saturate at 6 h in Arabidopsis, while in barley there was a steady increase for genes in these orthogroups up until 48 h (Figure 3). The genes in these orthogroups were associated with translation and ribosome biogenesis, indicative of activation of protein biosynthesis. Clusters 4 and 5 showed opposing co-expression profiles of genes in these orthogroups in the two species (Figure 3). Arabidopsis genes in these orthogroup clusters showed a transient decline in transcript abundances, while for barley the opposite was observed. For barley, genes in these two clusters were associated with enriched GO terms relating to seed germination, intracellular (protein) transport, and defense response. In contrast to these development-related GO terms in barley, for Arabidopsis, the GO term enrichment indicated that genes of orthogroups in clusters 4 and 5 are involved in the mobilization of nitrogen and, in particular, sulfur from protein catabolism, which might suggest seed storage degradation. Thus, consistent with the analyses above (Figure 3), when genes that are classified as orthologous were compared, both conserved and opposite patterns were observed. 

### 2.3. Opposite Transcriptome Patterns between Arabidopsis and Barley during Germination

**Figure 4:**
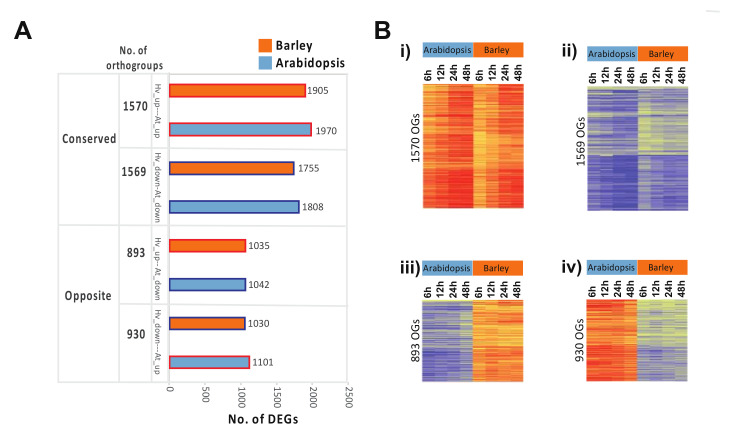
Expression of genes in orthogroups. (**A**) The number of DEGs in barley and Arabidopsis that were orthologous and showed conserved or opposite responses. Conserved was defined as orthogroups containing genes that were only up/downregulated over the time course at one or more time points in both species, while opposite orthogroups were identified as those in which opposite contrasting responses were observed between both species. Both groups exclude any orthogroups in which genes were both up- and downregulated over the time course and in which orthologues within the same orthogroup showed contrasting opposite responses for each species. (**B**) Heatmaps showing the conserved and oppositely responsive genes in barley and Arabidopsis, (i) genes in orthogroups that are conserved with an increase in transcript abundance in Arabidopsis and barley, and (ii) conserved with a decrease in transcript abundance in Arabidopsis and barley, (iii) genes in orthogroups that displayed a decrease in transcript abundance in Arabidopsis but an increase in barley, and (iv) genes in orthogroups that displayed an increase in transcript abundance in Arabidopsis but a decrease in barley.

To examine the orthologous gene responses more closely, we identified sets of genes that showed conserved or opposite responses in barley and Arabidopsis germination, excluding orthogroups in which genes were both up- and downregulated at different time points during germination (Figure 4). In this way, 1570 orthogroups were identified containing 1905 genes in barley and 1970 genes in Arabidopsis, showing a conserved upregulated response during germination (Figure 4A, Table S7A). Similarly, 1569 orthogroups were identified that contained conserved downregulated genes during germination (Figure 4A, Table S7B). 

In order to identify oppositely responsive orthologous genes, we isolated individual orthogroups in which transcripts were up/downregulated in barley, while their orthologous genes in Arabidopsis were oppositely responsive (Figure 4). Groups containing one or more transcripts that were responsive in the same up/downregulated manner between both species were also excluded (Table S7C,D). In this way, a set of 893 orthogroups were identified, containing 1042 Arabidopsis gene(s) that were downregulated at one or more time points during germination, whilst their 1035 barley orthologous gene(s) in the same respective groups were upregulated (Figure 4, Table S7C). Similarly, a set of 930 orthogroups containing 1101 Arabidopsis genes were identified to be upregulated at one or more time points during germination, whilst the 1030 barley orthologous gene(s) in the same respective groups were downregulated (Figure 4, Table S7D).

**Figure 5:**
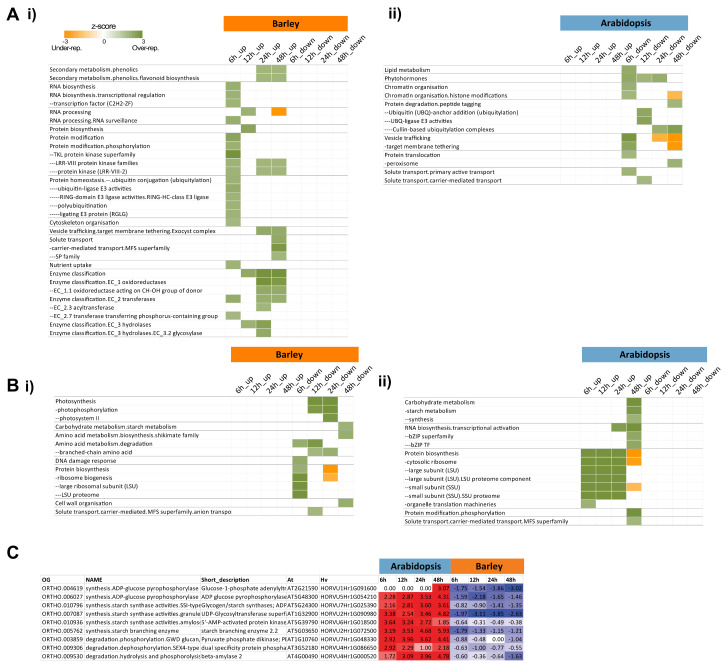
Analysis of genes showing the distinct responses during seed germination in barley and Arabidopsis. (**A**) The oppositely responsive gene sets in barley and Arabidopsis were analyzed for significantly over-represented functional categories (Fisher’s test, *p* < 0.05) using Pageman [37], and the outputs for the genes that were (i) upregulated in barley and orthologous to the (ii) downregulated genes in Arabidopsis are shown. (**B**) The significantly over-represented functional categories for the genes (i) downregulated in barley that were orthologous to the (ii) upregulated genes in Arabidopsis. (**C**) Orthologous genes encoding starch synthesis functions that showed opposing responses between barley and Arabidopsis.

Over-representation analyses of gene sets that displayed opposite patterns were carried out (Table S7C,D) (over-representation analysis (ORA) Fisher, *p* < 0.05, [37]). The upregulated gene set in barley and its respective downregulated Arabidopsis orthologous genes were analyzed separately and, as expected, similar functional categories were enriched (Figure 5Ai,ii, Table S8). Note that the functional annotations of genes differed between barley and Arabidopsis due to genome size, gene content, and the degree of annotation. The processes of ubiquitylation functions such as UBQ-ligase E3 activities, vesicle trafficking, and solute transport were significantly enriched in an opposite manner for both species (Figure 5Ai,ii). Similarly, when the over-representation analysis was carried out for the downregulated orthologous genes in barley that were upregulated in Arabidopsis (Figure 5Bi,ii), we identified protein biosynthesis functions (specifically ribosomal proteins); the major facilitator superfamily (MFS) of solute transporters; and carbohydrate metabolism, specifically starch metabolism, as being enriched in the downregulated genes in barley that were orthologous to upregulated genes in Arabidopsis (Figure 5Bi,ii). At a gene level, 41 DEGs in Arabidopsis that encode starch metabolism functions were differentially expressed, with nine of these genes belonging to orthogroups in which these were orthologous to genes that were downregulated in barley germination (Figure 5C). As barley is a starch seed while Arabidopsis is an oil seed, it may not be surprising to see differences in the response of genes encoding starch metabolism functions. However, these results do indicate that many other pathways that are not as obvious may also be differentially responsive between these species.

**Figure 6:**
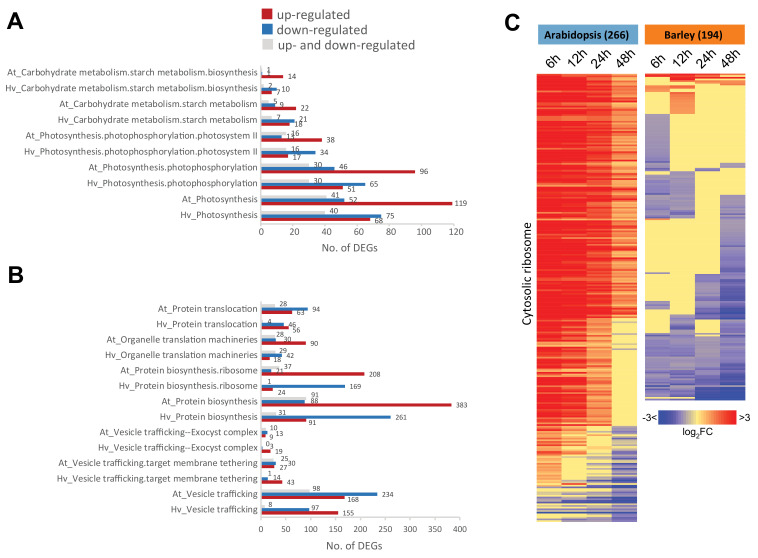
Analysis of distinct expression responses for energy- and protein-related functions during germination in barley and Arabidopsis. The over-represented functional categories in which more genes showed the opposite response to genes in the same category in Arabidopsis were identified, and the total number of DEGs that were upregulated, downregulated, and both up- and downregulated during germination are indicated for each functional category. (**A**) The energy-related functional categories in which more genes showed opposite responses. (**B**) The protein-related functional categories in which more genes showed opposite responses in barley and Arabidopsis. (**C**) Heatmap showing these responses for genes annotated as encoding cytosolic ribosome components.

**Figure 7:**
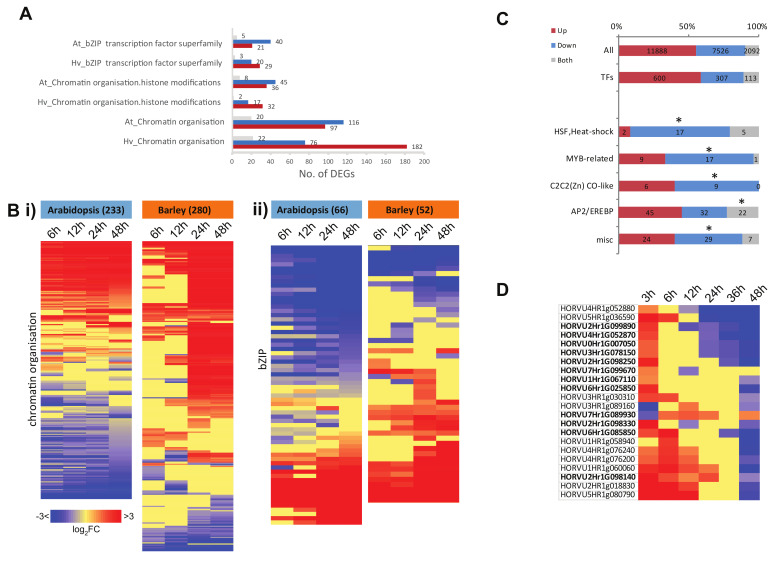
Expression of genes encoding DNA/RNA binding functions during germination. (**A**) The over-represented DNA/RNA binding-related functional categories in which more genes showed the opposite response to genes in the same category in Arabidopsis were identified, with the total number of DEGs that were upregulated, downregulated, and up- and downregulated during germination being indicated for each. (**B**) Heatmaps showing the expression of all genes encoding (i) chromatin organization components and (ii) basic leucine zipper (bZIP) TFs in Arabidopsis and barley. (**C**) Of the 1020 TFs identified, significantly enriched TF families in the up/downregulated genes as well as the genes that were both up- and downregulated during germination are shown (* *p* < 0.05). (**D**) The 22 genes encoding Apetala2 (AP2)/ethylene-responsive element binding proteins (EREBP) that were both up- and downregulated compared to 0 h during germination. Of these, 12 genes (indicated in bold) were identified from barely gene annotation only and not orthology.

To gain a more comprehensive outlook of these differences between species, we examined all of the genes in each of the significantly enriched functional categories identified in Figure 5A,B, irrespective of their orthology. Therefore, categories containing orthologous and non-orthologous genes for each species were examined. These were then filtered to identify only those categories in which more genes were up/downregulated in barley while more genes were responding the opposite way in Arabidopsis, and in this way a more stringent subset of the functions showing opposite responses between Arabidopsis and barley were discovered. This approach identified some pathways involved in energy functions (Figure 6A), protein/transport (Figure 6B), and DNA/RNA binding (Figure 7).

In addition to starch biosynthesis in the energy function categories, photosystem II components showed the same pattern, with more than twice as many upregulated genes (38 genes) as downregulated genes (13 genes) for these components in Arabidopsis, while the opposite occurred in barley with 34 downregulated genes and 17 upregulated genes (Figure 6A). For the protein/transport category, the most significantly over-represented functional category among the oppositely responsive DEG sets of Arabidopsis and barley was for protein biosynthesis functions, particularly for genes encoding ribosomal proteins, with 208 DEGs that were upregulated in Arabidopsis and 169 DEGs downregulated in barley (Figure 6B). These were predominantly made up of large subunit (LSU) and small subunit (SSU) ribosome protein components. When the fold changes of all 266 genes in Arabidopsis and 194 genes in barley that encode cytosolic ribosomal proteins were visualized (Figure 6C), we found that in Arabidopsis these genes peaked in expression early in germination (<12 h) and then decreased by 48 h. In contrast, the highest expression level for barley genes encoding ribosomal proteins was in the dry seed (0 h), with lower expression mostly observed during germination (Figure 6C). A similar pattern was also observed for genes encoding organelle translation machineries, including organellar ribosomal proteins (Figure 6B), with 90 genes encoding ribosomal proteins in organelles upregulated and 30 downregulated in Arabidopsis, compared to barley where only 18 genes encoding organelle translation machinery were upregulated compared to 42 downregulated. Note that the patterns observed here are consistent with the patterns observed for protein degradation, where the upregulation of protein synthesis functions in Arabidopsis is accompanied by a downregulation of protein degradation while in barley the opposite pattern is observed (Figure 5Aii).

The enriched functional categories involving DNA/RNA binding functions such as chromatin organization (specifically histone modification), as well as the bZIP family of TFs (Figure 5Aii,Bii), also had a greater number of genes that showed opposite responses (Figure 7A). When the fold changes for all genes encoding chromatin organization and bZIP TFs were visualized, we found that Arabidopsis had more downregulated genes compared to barley for genes in these categories/families (Figure 7Bii). For the genes encoding chromatin organization functions in Arabidopsis, we observed an early increase or decrease in abundance that remained throughout the time course of the analyses (Figure 7Bi). In barley, most of the genes displayed up- or downregulation only at the later timepoints at 24 h and 48 h (Figure 7Bi). 

A similar pattern was observed for bZIP-encoding genes, with nearly twice as many genes downregulated compared to upregulated in Arabidopsis, while the opposite was seen in barley, where more genes encoding bZIP TFs were upregulated, again at the later timepoints after 24 h (Figure 7Bii). In Arabidopsis, the bZIP TFs bZIP2 (At2g18160) and bZIP9 (At5g24800) have been shown to directly bind to the promoters of genes encoding proteins involved in the degradation of branched chain amino acid catabolism, activating alternative mitochondrial metabolism to promote survival when carbon sources may be limited [38]. bZIP11 (At4g34590) regulates carbon metabolism in a network with the sucrose non-fermenting-1 related protein kinase 1 (SnRK1) and trehalose 6-phosphate (T6P) [39]. bZIP44 has a direct role in Arabidopsis germination by binding to the promoter of an endo-β-mannanase and is involved in activation of alternative mitochondrial metabolism [38,40]. Together, this seems to indicate that despite the upregulation of carbon metabolism in Arabidopsis (Figure 5B), carbohydrate may still be limiting, and alternative energy metabolism is activated very early in germination until photosynthesis is established.

All genes encoding TFs in barley were examined to determine if any family was enriched in up-, down-, or both up- and downregulated gene sets (Figure 7C, Table S9A). Of the 1020 TFs identified, 600 genes were upregulated, 307 were downregulated, while 113 were both up- and downregulated at one or more time points over the course of germination (Figure 7C, Table S9B). When the occurrence of all TF families was examined in these sub-sets, we identified four TF families as significantly over-represented (*p* < 0.05) among the downregulated genes in barley, including heat shock factors (HSFs), my elob lastosis (MYB), CONSTANTs-like zinc finger family, and a group of miscellaneous TFs (Figure 7C, Table S8B). HSFs are also significantly enriched in the downregulated genes of Arabidopsis [41] and rice [22] seed germination, suggesting a potentially conserved role for these in the stored seed transcriptomes of these species. When the genes that were both up- and downregulated during barley seed germination were examined, we found AP2-EREBPs to be significantly enriched (Figure 7B). Specifically, these 22 genes peaked early in expression (mostly at 3 h post-imbibition) and decreased in expression as germination progressed (Figure 7C). Notably, the enrichment of AP2-EREBPs was also observed in the equivalent cluster in rice germination, peaking at 3 h post-imbibition [22].

### 2.4. Mitochondrial Dynamics during Barley Seed Germination

The energy to drive germination is derived from heterotrophic sources until photosynthesis can be established after imbibition. While mitochondria have been shown to play a critical role in germination in Arabidopsis [28,42,43,44] and seed aging, which is linked to changes in mitochondrial function [28,45], the role of mitochondria in Arabidopsis at a biochemical level has not been analyzed, simply due to the small size and ability to obtain sufficient mitochondria for analyses from embryos. This was possible in barley by using ≈700 embryos to obtain sufficient purified mitochondria for biochemical analyses, with 0.5 to 1.5 mg of mitochondria obtained from dry embryo to grains imbibed for 48 h. These analyses can also be compared to previous studies in rice and maize [21,22,23,25]. 

**Figure 8:**
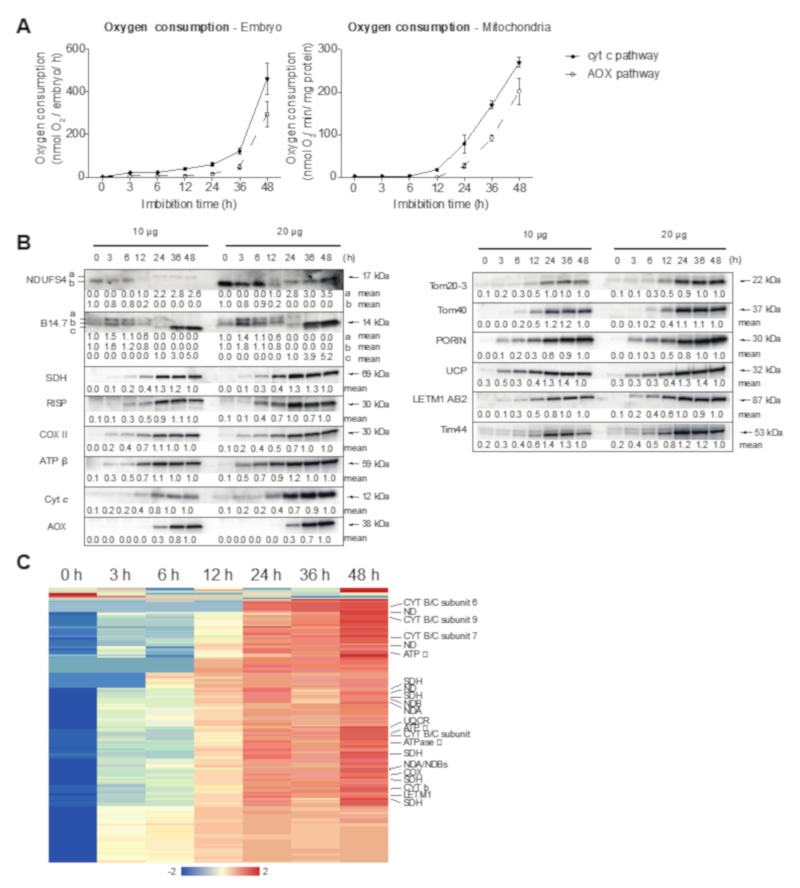
Mitochondrial dynamics during barley grain germination. (**A**) Oxygen consumption in whole embryos and isolated mitochondria. Oxygen consumption was examined in whole barley embryos and isolated mitochondria using a Clark-type oxygen electrode. Each data point represents an average of three to four independent measurements. (**B**) Changes in abundance of mitochondrial proteins. A representative image is shown from probing three independent isolated mitochondrial proteins using both 10 and 20 µg of isolated protein. The abundance of the protein is normalized to a value of 1 at 48 h and all other values are expressed in a relative manner. For NDUFS4 “a” and “b”, all values are relative to 12 h and 0 h, respectively. For B14.7 “a”, “b”, and “c”, all values are relative to 0 h, 0 h, and 24 h, respectively. The apparent molecular mass is indicated in kDa. NDUFS4 = 17 kDa subunit complex I, B14.7 = complex I subunit, SDH = subunit I of succinate dehydrogenase/complex II, RISP = Rieske FeS protein of complex III, COXII = cytochrome oxidase II of complex IV, ATPβ = β subunit of ATP synthase, Cyt c = cytochrome c, AOX = alternative oxidase, Tom20-3 = translocase of the outer membrane 20-3, Tom40 = translocase of the outer membrane 40, Porin = voltage-dependent anion channel, UCP = uncoupling protein, LETM1 = leucine zipper-EF-hand-containing transmembrane protein 1, Tim44 = translocase of the outer membrane 44. (**C**) The heatmap represents the abundance of 173 mitochondrial proteins at the indicated time points after seed inhibition. Quantification was performed by proteomic analysis (label-free quantification) of purified mitochondrial proteins, and abundances are represented by z-scored intensity-based absolute quantification (iBAQ) values. The position of several protein isoforms is indicated in the heatmap (see Table S10 for details): CYC B/C, CYTOCHROM b/c subunit; ND, NADH DEHYDROGENASE; SDH, SUCCINATE DEHYDROGENASE; NDA/NDB, ALTERNATIVE NAD(P)H DEHYDROGENASE; COX, CYTOCHROME OXIDASE; LETM1, LEUCINE ZIPPER-EF-HAND-CONTAINING TRANSMEMBRANE PROTEIN 1.

To gain insight into the role of mitochondria during barley germination, we examined their physiological, proteomic, and transcriptomic responses. Measurement of oxygen consumption in both whole embryos and isolated mitochondria showed a relatively slow increase over the first 6 to 12 h, but thereafter oxygen consumption increased substantially (Figure 8A). Specifically, in whole embryos and isolated mitochondria, oxygen consumption via the cyanide-sensitive cytochrome respiratory chain remained relatively low until 6 h after imbibition. From 6 to 36 h, a steady small rise in oxygen consumption was detected and thereafter the increase was much larger. In isolated mitochondria, a similar trend was observed, except that the substantial increase was detectable already after 12 h (Figure 8B).

The patterns of oxygen uptake are underpinned by the accumulation of proteins for the cytochrome respiratory complexes determined by immunoblot analyses. Quantification of the abundance of complex I subunit NDUFS4 (17 kDa subunit) and B14.7 complex I subunits [46], succinate dehydrogenase (complex II) subunit I (SDH) [47], Rieske FeS protein (RISP) complex III subunit [48], cytochrome oxidase subunit II (COXII) complex IV subunit [49], and the β-subunit of the ATP synthase complex V subunit [50] revealed that they increased in abundance up until 24 h and remained relatively constant thereafter (Figure 8B). With the exception of NDUFS4 and B14.7 subunits of complex I, all these subunits had reached about 50% of their maximum abundance 12 h after imbibition, coinciding with the large increase in oxygen consumption via the cytochrome pathway that was observed with isolated mitochondria. For NDUFS4 and B14.7, a complex pattern with multiple bands was observed, suggesting that there may be changes in isoforms that are expressed over development or in post-translational modification. For NDUFS4, isoform “b” with a slightly lower molecular mass was reduced from 0 h to 12 h, and not detectable after 12 h (Figure 8B). Then, isoform “a” started to be expressed from 12 h to 48 h (Figure 8B). For B14.7, three isoforms were detected over development. Isoform “a” and “b” were detected from 0 h to 12 h, with a transient induction at 3 h (Figure 8B). Isoform “c”, with a slightly lower molecular mass, was detected from 24 h to 48 h, with an at least threefold induction from 24 h to 36 h (Figure 8B). Protein abundance for the alternative oxidase (AOX) was not detectable until 24 h after imbibition in isolated mitochondria (Figure 8B). It was consistent with the lag in oxygen consumption via the alternative respiratory pathway observed to increase about 12 h later than the cytochrome pathway, and an increase in activity not observed until 12 to 24 h in isolated mitochondria and 24 to 36 h in whole embryos (Figure 8B). For proteins involved in mitochondrial biogenesis, an overall pattern of a steady increase in abundance to peak at 24 h was observed, i.e., for the translocase of the outer membrane 40 (Tom40) and Tom20-3 [51], translocase of the inner membrane 44 (Tim44) [52], and leucine zipper-EF-hand-containing transmembrane protein (LETM) [53]. Porin (voltage-dependent anion channel) and UCP (uncoupling protein) continued to increase in abundance up to 48 h post-imbibition (Figure 8B).Analyses of the mitochondrial proteome over germination by mass spectrometry gave a similar picture to the Western blot analyses described above (Figure 8C, Table S10): complex I (17 proteins detected), complex II (5 proteins detected), complex III (6 proteins detected), complex IV (1 protein detected), complex V (4 proteins detected), and cytochrome *c* all began to increase in abundance at 12 h and generally increased to reach a maximum at 36 to 48 h. This increase in protein abundance was in agreement with the detection of oxygen consumption at 12 h in isolated mitochondria that increased over time (Figure 8A). Some proteins were observed to increase in abundance at earlier time points. At 3 h, we observed two proteins involved in ubiquinone biosynthesis, a splicing factor, a single stranded DNA binding protein, a RNA binding protein, and the mitochondrial metalloendopeptidase OMA1 (overlapping with the *m*-AAA (matrix-**A**TPases **A**ssociated with diverse cellular **A**ctivities) protease) that has been associated with mitochondrial morphology by affecting fusion and fission that occurs early in germination in Arabidopsis mitochondria [26], as well as two iron-sulfur assembly proteins. This suggests that proteins involved in biogenesis increased earlier to facilitate the increase in oxidative metabolism that occurred later. 

**Figure 9:**
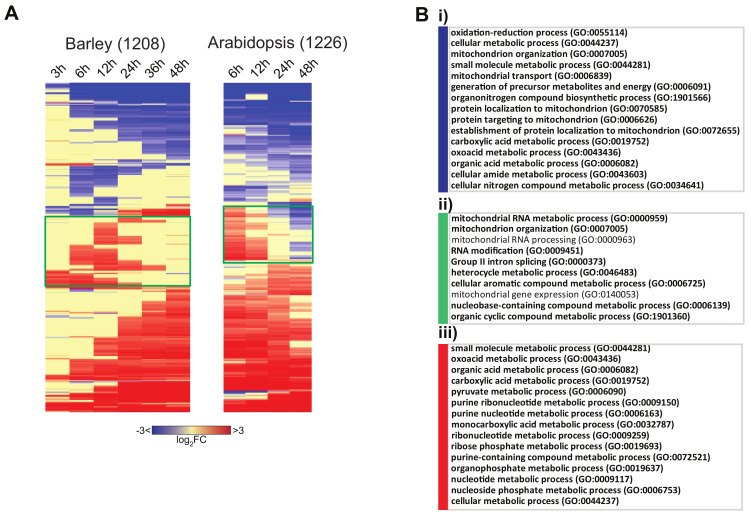
Expression of genes encoding mitochondrial proteins during germination. (**A**) Hierarchical clustering of the fold changes (logFC) compared to 0 h for the 1208 genes encoding mitochondrial proteins in barley and 1226 genes encoding mitochondrial proteins in Arabidopsis [10]. (**B**) GO over-representation analysis was carried out for the three clusters of responses in barley, and the top 15 significantly over-represented categories are shown, (i) for genes that decrease in transcript abundance during germination, (ii) for genes that display a transient increase in transcript abundance, and (iii) for genes that increase in transcript abundance during germination (Bonferronni; *p* < 0.05).

To gain insight into the regulation of barley genes encoding mitochondrial proteins, we hierarchically clustered 1208 genes encoding putative mitochondrial proteins (Figure 9A, Table S11A,B). When these genes were compared to the equivalent subsets in Arabidopsis [10], these genes peaked early during germination in both species, comprising ≈200 genes in Arabidopsis and 250 genes in barley (green boxes, Figure 9). Examination of the GO term enrichment revealed that at a functional level, transcripts of genes encoding mitochondrial proteins were conserved (Figure 9B). Consistent with the overall analyses of the transcriptome (Figure 2), the transiently peaking group of genes (Figure 9Bii, green) were enriched in RNA modification processes, which is likely associated with organelle-encoded gene expression. For the genes that decreased in transcript abundance, some processes associated with mitochondrial biogenesis were downregulated, such as protein localization to mitochondria, protein targeting to mitochondria, and establishment of protein localization to mitochondria (Figure 9Bi, blue). At a transcript level, this appears to be opposite of what is occurring at a protein level, whereby protein abundance (and activity) is increasing for the vast majority of functions (Figure 8). As expected, the transcripts encoding protein metabolism functions increased over time, underpinning both the biosynthetic and energetic role played by mitochondria in germination (Figure 9Biii). 

**Figure 10:**
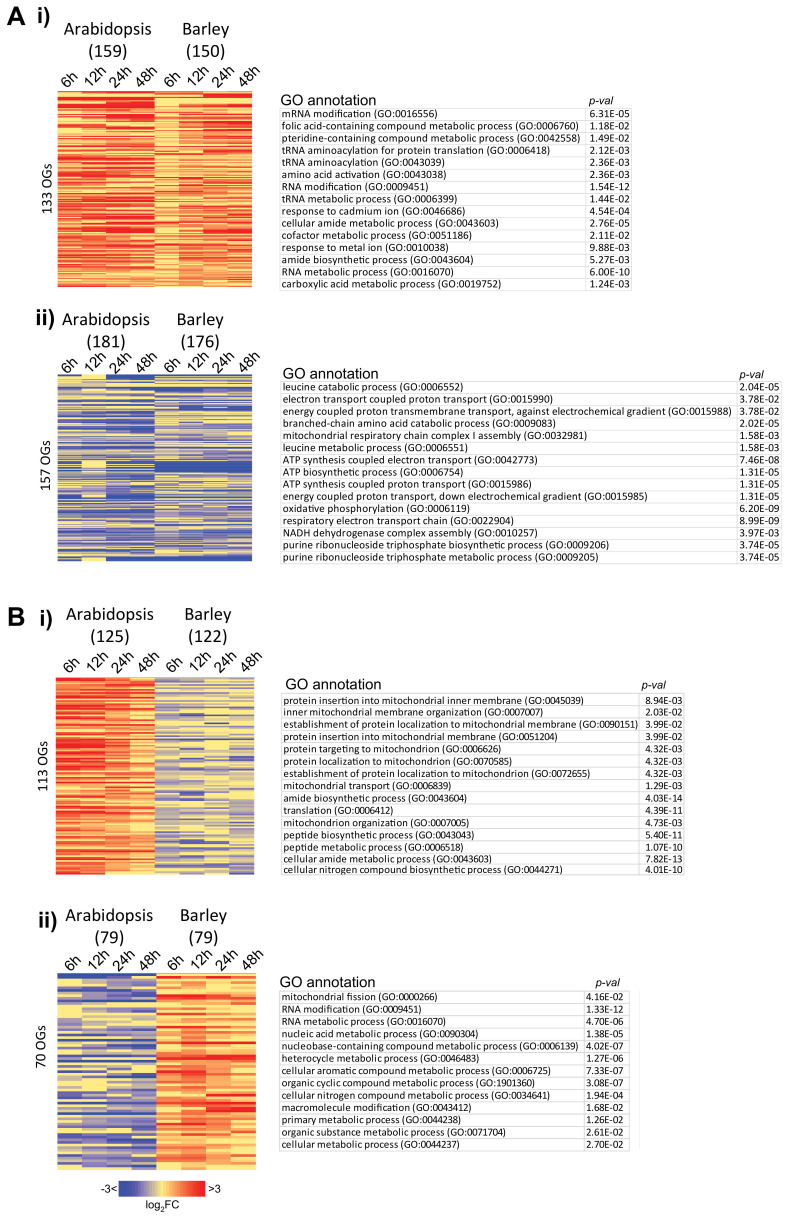
Analysis of genes encoding orthologous mitochondrial proteins that are responsive during germination in barley and Arabidopsis. (**A**) Heatmap showing the orthologous genes that showed conserved responses to germination in barley and Arabidopsis, with (i) conserved upregulated genes and (ii) conserved downregulated gene sets shown. The top 15 gene ontology-enriched categories are shown next to these (*p* < 0.05, Bonferroni). (**B**) Heatmaps showing orthologous genes that showed distinct responses to germination in barley and Arabidopsis, with (i) upregulated genes in Arabidopsis orthologous to downregulated genes in barley shown, and (ii) downregulated genes in Arabidopsis orthologous to upregulated genes in barley shown. The top 15 gene ontology (GO)-enriched categories are shown next to these (*p* < 0.05, Bonferroni).

In terms of orthology, 290 orthogroups that encode mitochondrial proteins in Arabidopsis and barley show the same trend, with 133 orthogroups displaying an increase in transcript abundance and 157 orthogroups displaying a decrease in transcript abundance (Figure 10A, Table S12A,B). Examination of the genes encoding these conserved trends showed that RNA metabolism (rRNA, mRNA, or transfer RNA (tRNA)) and biosynthetic processes are enriched in the conserved upregulated genes, while some processes associated with energy metabolism and ATP generation are downregulated (Figure 10A). As the latter is somewhat surprising given the increase in mitochondrial proteins associated with the electron transport chain (Figure 8), examination of the conserved downregulated list reveals that many of these genes are encoded in the mitochondrial genome. Consistent with what has been observed previously in Arabidopsis, the corresponding transcripts are already at high levels in dry seeds and in fact decrease in their abundance (Table S12B). Interestingly, 183 orthogroups displayed an opposite trend in transcript abundance (Table S12C), and as indicated previously, genes associated with mitochondrial protein localization appeared to be decreasing in barley germination but increased in Arabidopsis germination (Figure 10B). Six genes that encode proteins associated with protein import are annotated in this list, however, three of them (AT4G26670, AT5G55510, AT2G42210) do not have direct experimental roles in terms of being involved in protein import, but are nonetheless members of the family of the preprotein and amino acid transporters that are involved in protein import [54]. Translocases of the inner membrane (Tim8, 9, and 10), intermembrane proteins that have a role in import of mitochondrial carrier-type proteins, are listed, possibly indicating a difference in the metabolite exchange that takes place in different developing seeds (Table S12C) [55]. Interestingly, in the lists of orthogroups upregulated or with opposite expression in both species is a substantial number of genes that encode pentatricopeptide repeat (PPR) or tetratricopeptide repeat (TPR) proteins.

Thus, it appears at a functional level that mitochondrial biogenesis (i.e., an increase in mitochondrial protein abundance and function) is a common feature of seed germination (Figure 8), and while the overall functional annotation of genes seems highly conserved (Figure 9), at a gene level (Figure 10), the expression of genes suggests that there may be some differences in the functional state of mitochondria.

## 3. Discussion

Analysis of the transcriptome of barley during germination and comparison to Arabidopsis revealed that the developmental transcriptome from imbibition to the completion of germination displayed many conserved features. At an overall level, the transcriptome displays the same patterns: a relatively similar number of transcripts stored in the dry grain/seed; groups of transcripts that increase and decrease in abundance; and, importantly, a group of approximately 3000 transcripts that display both an increase and decrease in abundance over the relatively small period (Figure 2). Comparison of the respective transcriptomes using a strict orthology approach confirmed the conserved processes, with ≈4000 orthogroups displaying the same pattern of transcript abundance changes with several clusters showing the same pattern between the two species (Figure 3 and Figure 4). The fact that these conserved patterns were observed between Arabidopsis seeds that were cultivated under ideal conditions to produce seeds for germination analyses [10] and grain from field-grown barley that were stored for over one year before use, indicated that once imbibition occurs and germination commences, a well-conserved development program is initiated that is conserved over the 100 million years of evolution that separates monocots and dicots [31]. This pattern of transcriptome changes underlies the physiological changes that occur in both water and oxygen uptake that have been characterized over 50 years ago, with an initial rapid water and oxygen uptake (phase I), a plateau phase (phase II), and a further increase later to complete germination (phase III) [56].

However, 1800 orthogroups representing over 2000 genes were identified here that displayed opposite responses in transcript abundance between barley and Arabidopsis (Figure 4, Table S4C,D). Functional categories of metabolism (carbohydrate, lipid, and secondary metabolism), photosynthesis, protein synthesis, modification and degradation (protein degradation ubiquitin, protein synthesis ribosome), and regulation (chromatin organization, TFs) were notable as important processes for growth and development that were over-represented among these oppositely responsive genes (Figure 5, Table S7). At least some of these differences are likely related to the difference between the small oil storing Arabidopsis seed and the larger starch storing barley seed, however, these differences are important to reveal as they may provide opportunities for translation of knowledge or processes between different systems and may give insight into where regulatory processes have diverged.

Initially on the basis of GO and orthology, we observed that there was a difference in the pattern of transcript abundance for carbohydrate-related functions (Figure 5B,C). Analyses of all genes associated with carbohydrate and photosynthesis showed that for several categories, Arabidopsis had twice as many genes that were upregulated in abundance compared to being downregulated. In contrast, barley had more downregulated genes for these functional categories and a smaller number of upregulated genes compared to Arabidopsis. While some differences in starch metabolism would be expected between starch and oil storing seeds, the fact that photosynthesis-associated genes show a similar opposing trend suggests that it is not just a simple re-routing of carbohydrate versus oil metabolism. It appears that the machinery to underpin autotrophic growth is activated at a more rapid pace in Arabidopsis than in barley, which may reflect the amount of stored reserves rather than the type of reserve. While no previous analyses of barley germination over this time course exists, a previous study examining barley seed maturation and germination did show that photosynthesis was upregulated at 24 h and at a later time in barley, consistent with the study here wherein photosynthesis was established later in barley than in Arabidopsis [12]. The small seeds of Arabidopsis may have a limited capacity to support heterotrophic growth and thus there is an immediate upregulation of the machinery required to support autotrophic growth. Another difference that was apparent with respect to energy metabolism is that in Arabidopsis, the mitochondrial alternative energy pathway was activated, which uses the breakdown of amino acids to drive energy production, indicating that at the very early stages of germination, Arabidopsis may be carbon-limited and amino acid oxidation may play an important role in providing energy. Furthermore, a variety of bZIP TFs that have previously been shown to regulate this pathway are upregulated in Arabidopsis, supporting a possible very early activation of this pathway (Figure 7) [38,40]. A study in *Brassica napus* examining germination rate pointed at the level of the metabolites malate and asparate as markers for the rate of germination [27], as well as the extensive protein–protein links between the TCA cycle and amino acid metabolism [57], showcasing the rapid activation of carbon and nitrogen metabolism as important features in oil seeds for rapid germination. 

As mitochondria play a crucial role in metabolism, and the pattern of oxygen uptake appears to be conserved in seed germination, it would be expected that the processes of mitochondrial function would also be well conserved. Examination of the profile of transcript abundance for mitochondrial proteins reveals that 38 out of 40 functional categories were conserved over the time course of germination (Figure 9), compared to 9 out of 45 for the whole transcriptome (Figure 2C). Additionally, 400 orthogroups containing genes encoding mitochondrial proteins displayed a conserved pattern of up- or downregulation of abundance during germination (Table S11A,B). Nevertheless, some differences were also observed including those for certain genes whose transcript abundances were downregulated in Arabidopsis but whose orthologue(s) were upregulated in barley, with these encoded proteins being associated with mitochondrial DNA repair (At3g20540, At1g50840 polymerase gamma), 25 PPR or TPR proteins, lipid synthesis (DGD1_UDP-glycosyltransferase, cardiolipin synthase), dynamin-related proteins 3A and 3B, and elongated mitochondria1 (ELM1) involved in mitochondrial fission ([58] and DGS1 (dgd1 suppressor 1), which is involved in both lipid and protein biogenesis in plant mitochondria [59]. This suggests that these processes may be already established in Arabidopsis, while in barley they are still actively occurring. Genes whose transcript abundance was upregulated in Arabidopsis, but whose orthologue(s) were downregulated in barley, encoded proteins including a variety of ribosomal proteins, mitochondrial substrate carrier proteins, and antioxidant systems such as manganese superoxide dismutase (Table S11C,D). Combining these differences suggests that Arabidopsis may be more active or have more capacity for activity compared to barley, with parts of the antioxidant system already expressed. Direct analyses of genes encoding mitochondrial components in barley showed a gradual increase in transcript abundance over time that continues to increase until 48 h (Figure 8). While there is no direct data to compare to Arabidopsis, this does differ to previous studies in rice and maize. In rice, it was shown that proteins such as Tom20 and Tom40 were higher in abundance at 0 h (dry seed) compared to 48 h [21], which is clearly different to that observed here in barley. Likewise, the abundance of mitochondrial-encoded subunits in maize and rice was already high at the early stages in germination, as was porin [21,23], compared to in barley where it steadily increased over time to peak at 24 or 48 h. Moreover, overall, organelle translation machineries were upregulated in Arabidopsis compared to barley (Figure 5B), suggesting that mitochondrial capacity was overall increasing rapidly in Arabidopsis. Given the important role of components involved in mitochondrial biogenesis for the timing of germination, such as Tim17-1 ([44], and overall the importance of mitochondrial function for germination [14,60,61,62]), the kinetics of mitochondrial biogenesis during germination may play an important role in determining the speed of germination. 

One of the most striking differences observed between barley and Arabidopsis was the difference with respect to genes encoding protein synthesis functions (Figure 6B,C). In Arabidopsis, the genes encoding proteins involved in protein synthesis functions were upregulated in abundance, with almost 600 upregulated genes in the two categories of protein biosynthesis in Arabidopsis, compared to less than 100 in barley, while over 400 genes in this category were downregulated in barley. As pointed out above, a similar pattern was observed with organelle translation machinery. Given that ribosomes and protein synthesis represent significant investment in resources, the correlation of ribosome abundance with growth rate in unicellular organisms, and the additional cost of protein turnover in multi-cellular organisms ([63] and references therein), the differential investment in protein synthesis machinery during germination between Arabidopsis and barley indicates that different strategies may be pursued to achieve germination. This high demand for energy may also account for the activation of the alternative mitochondrial energy pathway in Arabidopsis compared to barley, as outlined above. It was notable that a group of 22 AP2/EREPB TFs displayed upregulation in abundance at the earliest stages of germination (3 to 12 h) and downregulation in transcript abundance at the later stages of abundance (24 to 48 h) in barley (Figure 7D), as well as the fact that AP2 TFs are associated with responses to hypoxia [64]. It has been shown for both root and shoot meristems that hypoxia plays an important role in development, with the N-end rule determining the stability of TFs to activate a hypoxic response [65]. The upregulation of AP2 factors at the start of germination may indicate that the larger barley grain is under hypoxic conditions after imbibition and at the earliest stages of germination. The role of ethylene-responsive element binding factor (ERF)-type TFs in cereals relating to both hypoxia and ABA regulation has been studied in cereals [66], and ABI5 has been shown to play a role in seed maturation and germination in wheat [67]. Thus, when comparing germination and functions of the genes involved in germination between plant species, it is worth noting that whilst the responses of many genes are highly conserved in the basic pattern of expression over the time-course, there are notable differences whereby orthologous genes can show opposite expression patterns, indicating possible differences in regulation.

## 4. Materials and Methods 

### 4.1. Plant Materials

Dehulled grains of *Hordeum vulgare* were germinated in the dark for 3, 6, 12, 24, 36, and 48 h at 22 °C on moist clotting paper in Petri dishes. Embryos were manually dissected from grains imbibed for different time courses, including 0 h (dry seeds).

### 4.2. Mitochondrial Isolation

Embryos (700−900) were dissected from dry or imbibed barley grains and subsequently used to isolate mitochondria according to the protocol described previously [68]. Isolated embryos were ground at 4 °C in grinding buffer (0.3 M sucrose, 25 mM tetrasodiumpyrophosphate, 2 mM EDTA, 10 mM KH_2_PO_4_, 1% (w/v) PVP-40, 1% (w/v) bovine serum albumin (BSA), 6 mM sodium ascorbate, and 5 mM L-cysteine (pH 7.5)) using a mortar and pestle. The homogenate was filtered through four layers of miracloth. The filtered homogenate was centrifuged at 2500× *g* for 8 min and the resulting supernatant was centrifuged at 17,500× *g* for 20 min. The pellet was re-suspended in wash buffer (0.3 M sucrose, 10 mM N-Tris (hydroxymethyl)-methyl-2-aminoethanesulfonic acid (TES), 0.1% (w/v) BSA (pH 7.5)) using a small paintbrush. Subsequently, samples were centrifuged at 2500× *g* for 8 min and the resulting supernatant was centrifuged at 17,500× *g* for 20 min. The pellet was re-suspended in wash buffer and layered on a continuous density gradient and centrifuged at 40,000× *g* for 40 min. The mitochondrial fraction that appeared at the bottom of the tube was transferred to a new tube and washed twice in wash buffer without BSA.

### 4.3. Water Content Measurement

Twenty embryos dissected from barley grains were immediately weighed (wet weight, WW) and then dried at 65 °C for 24 h; then, we reweighed (dry weight, DW) the dry embryos after putting them in a desiccator at 25 °C for 30 min. Measurements were carried out with three biological replicates at each time point. Water content (%) = (WW − DW)/DW × 100%.

### 4.4. Respiratory Measurements

Oxygen consumption measurements were conducted using a Clark-type oxygen electrode at 25 °C. For respiratory measurements on embryos, we added 10 embryos in 2 mL of respiration buffer (50 mM HEPES, 10 mM MES, 0.2 mM CaCl_2_ (pH 6.6)). For respiratory measurements on isolated mitochondria, we suspended 100 μg of mitochondrial protein in 1 mL respiration buffer (0.3 M sucrose, 5 mM KH_2_PO_4_, 10 mM TES, 10 mM NaCl, 2 mM MgSO_4_, and 0.1% (w/v) BSA (pH 7.5)). The following reagents were added to respiration buffer to initiate or inhibit reactions: ATP (0.3 mM), succinate (5 mM), ADP (0.25 mM), NADH (1.5 mM), myxothiazol (5 μM), n-PG (5 μM).

### 4.5. Immunoblot Analysis

Mitochondrial proteins were separated by SDS-PAGE and transferred to Hybond-C extra nitrocellulose membranes. Immunodetections were performed as described previously [52] with three biological replicates. To ensure linearity of the response of antibodies, we loaded two dilutions (10 μg and 20 μg) of total mitochondrial protein on the SDS gel. The intensity of cross-reacting bands was analyzed using the Image Lab software (Bio-Rad, Sydney, Australia). The pixel densities were expressed relative to 48 h after imbibition, with the pixel density adjusted to 1. For NDUFS4 and B14.7, the pixel densities were normalized to 1 at the time point when the isoform starts to be expressed. Antibodies were used against Porin [69], AOX [69], UCP [70], LETM1 [53], Tom20 [51], RISP [71], COXII (Agrisera, Vännäs, Sweden Cat #AS04 0543A), cytochrome *c* (Agrisera Vännäs, Sweden, Cat # AS08 343A), β-subunit of ATP synthase (Agrisera Vännäs, Sweden, Cat # AS05 085), and Tim44 [52]. For NDUFS4 (AT5G67590) and SDH1-1 (AT5G66760) antibody, we cloned full-length cDNA into pDest17 using Gateway technology and transformed it into the *Escherichia coli* BL21-AI (DE3) expression strain according to the manufacturer’s instructions (Invitrogen, Sydney, Australia). Moreover, recombinant proteins were expressed and purified by denaturing immobilized metal affinity chromatography (IMAC) using the Profinia protein purification system (Bio-Rad, Sydney, Australia). Recombinant proteins were confirmed by mass spectrometry prior to inoculation into rabbits. Three aliquots of 200 µg purified protein were used for injection into rabbits (WEHI, Australia) and the serum was tested by immunoblot.

### 4.6. RNAseq Analysis

Total RNA was isolated from dry or imbibed barley seed embryos. RNAseq was carried out for four biological replicates. Total RNA was extracted using the Spectrum Plant Total RNA kit (Sigma-Aldrich, Sydney, Australia) and treated with on-column DNase. The quantity and quality of RNA was analyzed using a SPECTROstar (BMG LABTECH, Freiburg, Germany) spectrophotometer and agarose gel electrophoresis. 

RNAseq libraries were prepared using the TruSeq Stranded mRNA Library Prep Kit according to the manufacturer’s instructions (Illumina, Melbourne, Australia) and sequenced on a NextSeq500 system (Illumina, Melbourne, Australia) as 75 bp reads with an average quality score (Q30) of above 90% and on average 22M reads per sample. Quality control was performed using the FastQC software (https://www.bioinformatics.babraham.ac.uk/projects/fastqc/). Transcript abundances as transcripts per million (TPM) and estimated counts were quantified on a gene level by pseudo-aligning reads against a k-mer index build from the representative transcript models (IBSCv2 for barley, TAIR10 for Arabidopsis, IRGSP-1.0 for rice) using the kallisto program with 100 bootstraps [72]. Only genes with at least 5 counts in a quarter of all samples per time point were included in the further analysis. The program sleuth with a likelihood ratio test was used to test for differential gene expression [73]. Genes were called as differentially expressed with a false discovery rate (FDR) < 0.05 and a |log_2_ (fold change) |>1. RNAseq data were deposited to the NCBI (National Center for Biotechnology Information) Sequence Read Archive under project number PRJNA577343.

### 4.7. Bioinformatic Analyses

For hierarchical clustering and generation of heat maps, we used the Partek Genomics software suite version 6.16 (Partek Incorporated, http://www.partek.com/). Orthologues and corresponding orthogroups among barley, Arabidopsis, and rice were inferred via OrthoFinder v. 2.3.3 (https://github.com/davidemms/OrthoFinder) with default parameters and MMseqs2 [74] for sequence similarity searches. Protein sequences for this were from EnsemblPlants v44 for barley (IBSCv2) and TAIR (TAIR10 release) for Arabidopsis. Co-expression analysis of orthogroups was performed using the Clust software v1.10.7 [36] with default parameters. 

For the TF analyses, the list of 1020 TFs was generated by first identifying the 887 TFs defined by annotation using the Mercator pipeline [75] and adding another 133 putative TFs on the basis of their barley gene descriptions. These were then classified into families and a z-score test was carried out to identify any significantly over-represented families (*p* < 0.05).

For the analysis of genes encoding mitochondrial proteins (as shown in Figure 9 and Figure S1), the lists of mitochondrial proteins for barley, rice, and Arabidopsis were generated using SUBA4 [76] and Croppal [77] with subsequent manual curation. Criteria included sequence-based prediction of mitochondrial proteins as well as confirmatory evidence from the literature for Arabidopsis, rice, or barley.

### 4.8. Proteomic Analysis 

The purified mitochondrial proteins were identified by LC–MS/GC–MS and quantified with label-free quantification method using MaxQuant. 160517_Hv_IBSC_PGSB_r1_proteins_HighConf_REPR_annotation.fasta.gz 01-Jun-2016 22:04 9.1M was downloaded from (http://webblast.ipkgatersleben.de/barley_ibsc/downloads/) and used as the database for the search engine. Protein identification was performed using the proteomics search engine Andromeda with the following parameters. Variable modifications: oxydation (M), acetylation (protein N-term); fixed modifications: carbamidomethyl (C); digestion: Trypsin with max 2 missed cleavages; minimum peptide length for specific cleavage: 7; maximum peptide mass: 4600 Da; minimum peptide length for unspecific cleavage: 8; maximum peptide length for unspecific cleavage: 25; FDR for PSM and protein: 0.01. Label-free quantification was performed with the following parameters. Match between runs were performed with a match window of 0.7 min and large label-free quantitation method (LFQ)-derived ratios were stabilized to reduce the sensitivity for outliers. In addition to LFQ, intensity-based absolute quantification (iBAQ)-derived values were also calculated.

## 5. Conclusions

In this study, the transcriptome changes during germination were analyzed for non-dormant barley grain and Arabidopsis seeds, as both the used seed transcriptome sets displayed almost 100% germination. The opposite patterns in the observed transcriptomes indicated that there is more than one roadmap to achieve germination. The reason for the observed differences could be due to several reasons, including the type of reserve stored, the amount of reserve stored, differences in development, and differences in oxygen availability. While it is not possible to distinguish between these possibilities and other unknown differences, the knowledge that there are differences allows translation of these processes between species to achieve desirable traits in seed germination. 

## Supplementary Materials

Supplementary materials can be found at https://www.mdpi.com/1422-0067/21/19/7404/s1.
