# Supplementary material for: Conserved and Opposite Transcriptome Patterns during Germination in Hordeum vulgare and Arabidopsis thaliana
Source: Int J Mol Sci. 2020 Oct 7;21(19):7404. doi: 10.3390/ijms21197404 (PMC7584043; doi:10.3390/ijms21197404)
Supplement: Supplementary file 1 [file ijms-21-07404-s001.zip › Supplementary Files/Supplemental Figure 2 V5.8.pdf]

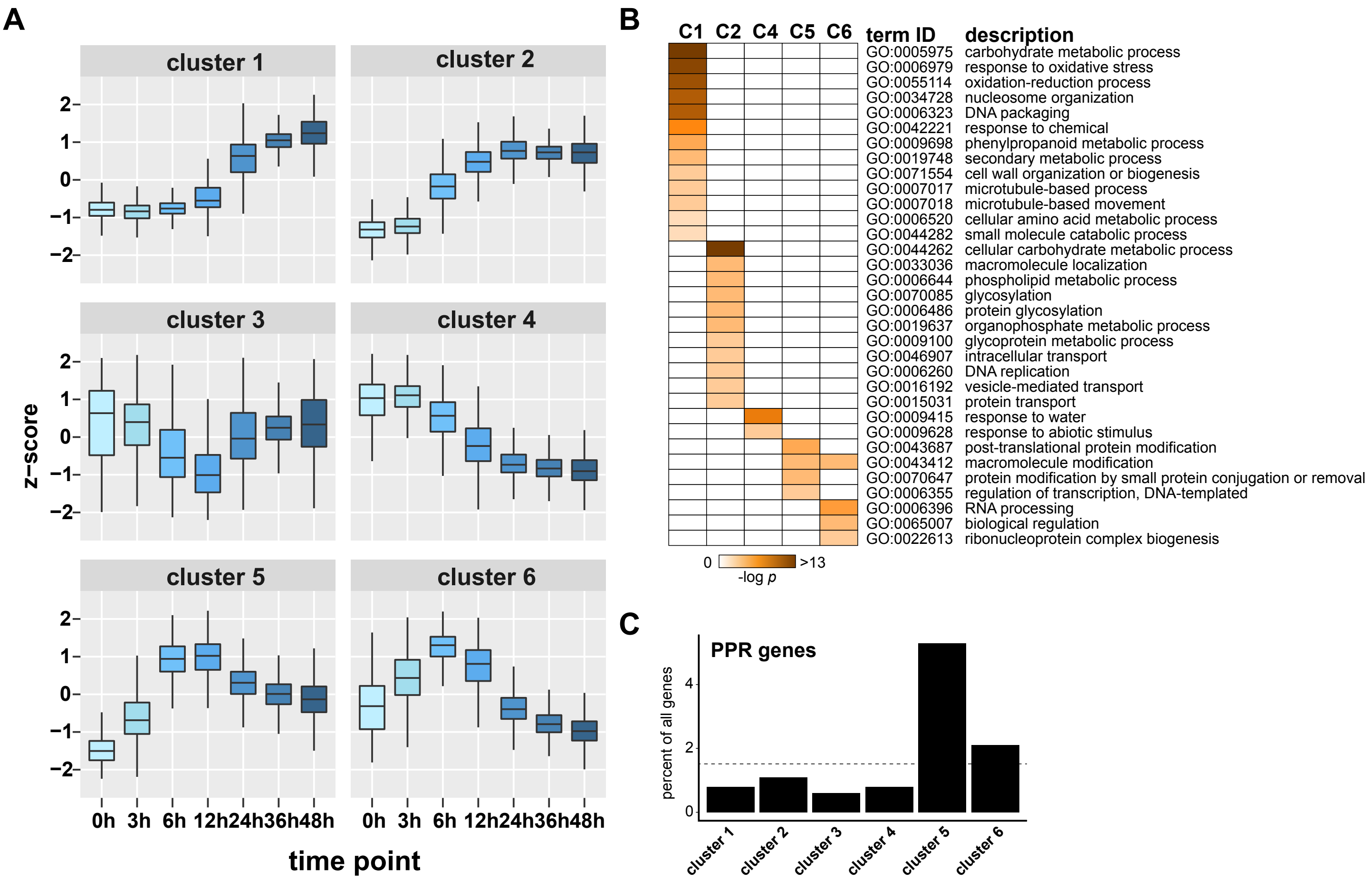

Supplemental Figure 2. Time course of expression of differentially expressed genes. **A)** Genes that were defined as differentially expressed across the time course compared to 0 h were clustered using a self-organising map algorithm. This identified 6 clusters with distinct expression patterns. For clusters 1 and 2 genes showed increasing expression over time and are enriched in GO terms (B) that would be expected for growth such as ‘carbohydrate metabolic process’, ‘cell wall organisation or biogenesis’. Genes in cluster 4 had decreasing transcript abundances with the GO terms ‘response to water’ and ‘response to abiotic stimulus’, likely representing processes related to the uptake of water. Clusters 5 and 6 display an initial increase in transcript abundance followed by a decrease and thus represent the transient group of genes, with the GO terms such as ‘regulation of transcription’, ‘RNA processing’ and ‘biological regulation’ enriched. The peak in transcript abundance for these clusters took place at or before 12 h and is similar in pattern and GO term enrichment to what has been previously observed for Arabidopsis [10, 41]. In Arabidopsis a group of several thousand transcripts enriched in components required for germination and mitochondrial biogenesis display a transient increase in abundance early in germination and contain a large number of pentatricopeptide repeat (PPR) proteins [10,41]. Analysis of the transient clusters 5 and 6 revealed that transcripts of genes encoding PPR proteins were much more prevalent in cluster 5 and 6 compared to all other clusters (C). In these two clus - ters are also genes encoding components of the mitochondrial protein import apparatus (TOM40, TIM23, TIM50, SAM50, LETM, HSPs, Chaperonins), mitochondrial DNA and RNA processing (RNA Helicase, Glutamyl-tRNA amidotransferase, Mitochondrial transcription termination factor family proteins (MTERFs), DNA mismatch repair protein mutS, intron maturase, Ribosome maturation protein SBDS, DNA and RNA polymerase, topoisomerase) as well as proteins involved in lipid, nucleotide and heme synthesis. This suggest that mitochondrial biogenesis represent a common and early process that occurs in germination. **B)** GO term analysis for all genes and for genes in clusters 5 and 6 that displays a transient pattern of expression. **C)** Percentage of pentratricopeptide protein (PPR)- coding genes in the six clusters. The dotted lines give the percentage of genes encoding PPR proteins in all clusters.
